# Leading people at different stages of personal development: an actionable framework for practitioners balancing emotional support and growth for a conscious future

**DOI:** 10.3389/fpsyg.2025.1636798

**Published:** 2025-11-26

**Authors:** Markus M. Luedi, Susan Goldsworthy

**Affiliations:** 1 Department of Anesthesiology Rescue- and Pain Medicine, Cantonal Hospital of St. Gallen, St. Gallen, Switzerland; 2 Department of Anesthesiology and Pain Medicine, Inselspital, Bern University Hospital, University of Bern, Bern, Switzerland; 3 International Institute for Management Development IMD, Lausanne, Switzerland

**Keywords:** personal development, secure base leadership, emotional intelligence, psychological growth, care–challenge balance, integral theory

## Abstract

Leadership is no longer just about directing tasks or achieving short-term goals. It is about navigating complexity, fostering growth, and addressing the human dimension of work in an era of constant change. Today’s leaders are expected to balance emotional support with strategic challenges, a dynamic process that empowers teams to thrive while fostering personal development. However, effectively guiding individuals at different stages of personal growth remains challenging. We present a conceptual synthesis that integrates insights from developmental psychology, integral theory, philosophy, neuroscience, and contemporary leadership concepts (e.g., authentic, adaptive, transformational, and servant leadership) to explore the transformative concept of “secure base leadership.” The originality of this paper lies not in surveying a wide range of thinkers, but in integrating the core strands into a coherent framework for leadership across stages of personal development.

## A world of paradoxes

Leadership is a tremendously complex concept that involves oneself, other individuals, groups, society, and in today’s reality even global communities. Unlike management that is following rules and thus predictable avenues, leadership deals with paradoxes, often arising between individual and organizational needs, that cannot be simply managed (Luedi, 2022). The successful resolution of these paradoxes has always been at the very core of leadership (Luedi, 2022), and with maturing societies, leadership became increasingly inclusive and cannot be described in single and thus segregated models anymore.

### Historical trajectories of leadership

The question about how individuals bridge between opposing forces within a system to help groups coalesce around shared goals is as old as mankind. Throughout history, humanity is a story of how both individuals and groups have organized themselves to solve problems, achieve common goals, and survive in a complex world (Sachs, 2012; Laloux, 2014). Thereby leadership has evolved alongside human societies reflecting the distribution of power, which itself has always evolved by technological, economic, and subsequent cultural changes and needs (Sachs, 2012; Laloux, 2014). The first concepts of leadership emerged naturally from small, task-oriented hunter-groups where leaders were often the most skilled, physically strongest or most knowledgeable individuals (Laloux, 2014). With the rise of agriculture and permanent settlements, leadership became formalized and societies developed hierarchies, with leadership roles often passed down through families (hereditary monarchies) or more democratic (ancient Greece), whereby the latter was reinforced in the renaissance (Russell, 2004). With the following industrial revolution, managing factories, workers, and growing urban populations was achieved by bureaucracy and rational-legal authority (Russell, 2004).

### From management to meaning: the shift in focus to resolve paradoxes

In the 2^nd^ half of the past century, the concentration camp survivor and psychiatrist Viktor Frankl developed the psychotherapeutic theory of “logotherapy,” i.e., an individual’s (mindful) search for personal meaning (Frankl, 1985). At the end of the last century, Daniel Goleman’s milestone article on emotional intelligence (Michel and Neuman, 2010) paved the way for systematically integrating mindfulness into leadership practice and to thereby embodying the experience of interdependent relationships and relational growth beyond the “ego” in professional environments, demonstrated in practice by leaders such as Mandela (2008). This understanding of leadership allowed for an ever increasing focus on purpose, vision and empowering others rather than controlling them (Smith, 2009). While contemporary leadership studies build on this historical trajectory with theories such as authentic, adaptive, transformational, and servant leadership, philosophical traditions highlight humanity’s enduring search for meaning and connection.

We present a conceptual synthesis that integrates insights from psychology, philosophy, and leadership practice toward a theoretically grounded and actionable framework for leadership practitioners. The originality of our argument will not lie in surveying them all, but in integrating three core strands: developmental psychology (Maslow, 2013; Jung, 2014), integral theory (Wilber, 1997) and secure base leadership (Kohlrieser et al., 2012). Together, they provide a coherent framework for linking personal growth with leadership behaviors across stages of personal development. While our synthesis’ validity rests on the coherence of arguments and the bridging of existing theories with practitioner-oriented frameworks, it develops the integrative model “secure base leadership” across different stages of personal development. Therefore, rather than to provide primary data, our intention is to offer a coherent synthesis that stimulates further empirical inquiry.

### Verb-based frameworks for leadership transformation

Studying expert organizations, Laura Empson coined the term “insecure overachievers” to describe typical leaders, especially in expert organizations (Empson, 2017). Empson’s concept highlights how individuals who excel in managing knowledge and executing tasks, or simply “doing” and “knowing” are sustaining productivity and their careers with socially accepted behavior. From a psychotherapeutic perspective (Freud, 1989), these individual experts disconnect from the authentic self to fight the, socially allegedly unacceptable, personal insecurity by individually chasing measurable achievements and external validation based on the organization’s need with relentless dedication. While the easily quantifiable values “doing” and “knowing” are widespread in organizations globally, such focus on tangible behavior (“doing”) and intellectual mastery (“knowing”) can drive burnout and can hinder personal growth and resilience and building genuine, trust-based relationships, or simply put, “being” and “connecting.” This transformation is crucial for fostering more sustainable leadership and requires continuous personal development of one’s identity. Interestingly, different contemporary leadership theories offer verb-based concepts: Theory U for example takes individuals (and organizations) through a U-shaped journey of letting go, presencing, and bringing forth new realities (Scharmer, 2016). Scharmer frames this with verbs to emphasize lived processes rather than fixed states: “Knowing” as embodied awareness beyond habitual thinking, “connecting” as empathetic relationship with others and the larger whole, “being” as presencing and alignment with deeper purpose, and “creating” as crystallizing and prototyping emerging futures (Scharmer, 2016). Similarly, Peter Senge’s model of a learning organization highlights the interplay of verbs: “being/knowing” through personal mastery, and “connecting/doing” through a shared vision and team learning (Senge, 1996). In the same spirit, the United Nation’s (UN) Inner Development Goals initiative frames leadership as cultivating inner capacities, balancing “knowing,” “connecting,” “being,” and “creating” to support achievement of the UN Sustainable Development Goals (Ankrah et al., 2023). Together, these frameworks underscore a shift in leadership thinking from static traits toward dynamic, verb-based capacities that enable transformation in complex environments.

Authentic leadership (George, 2003), with roots in positive psychology (Avolio and Gardner, 2005) emphasizes self-awareness, values, and integrity, aligning closely with the “being” dimension of leadership (George, 2003; Avolio and Gardner, 2005). Adaptive leadership stresses mobilizing people to address complex challenges by shifting perspectives and learning, resonating with “knowing” and “connecting.” (Heifetz, 1994; Linsky and Heifetz, 2002) Transformational leadership inspires followers to transcend self-interest through vision and purpose, reflecting the “connecting” and “being” dimensions (MacGregor, 1978; Bass and Riggio, 2006). Servant leadership highlights prioritizing the growth and well-being of others, echoing the “caring” side of secure base leadership (Greenleaf, 1998). Goleman’s six emotional-intelligence based styles - coercive, authoritative, affiliative, democratic, pacesetting, and coaching - provide practical manifestations of how leaders can flexibly balance “caring” and “daring” in context (Goleman, 2017).

### Secure base leadership

**Figure 1:**
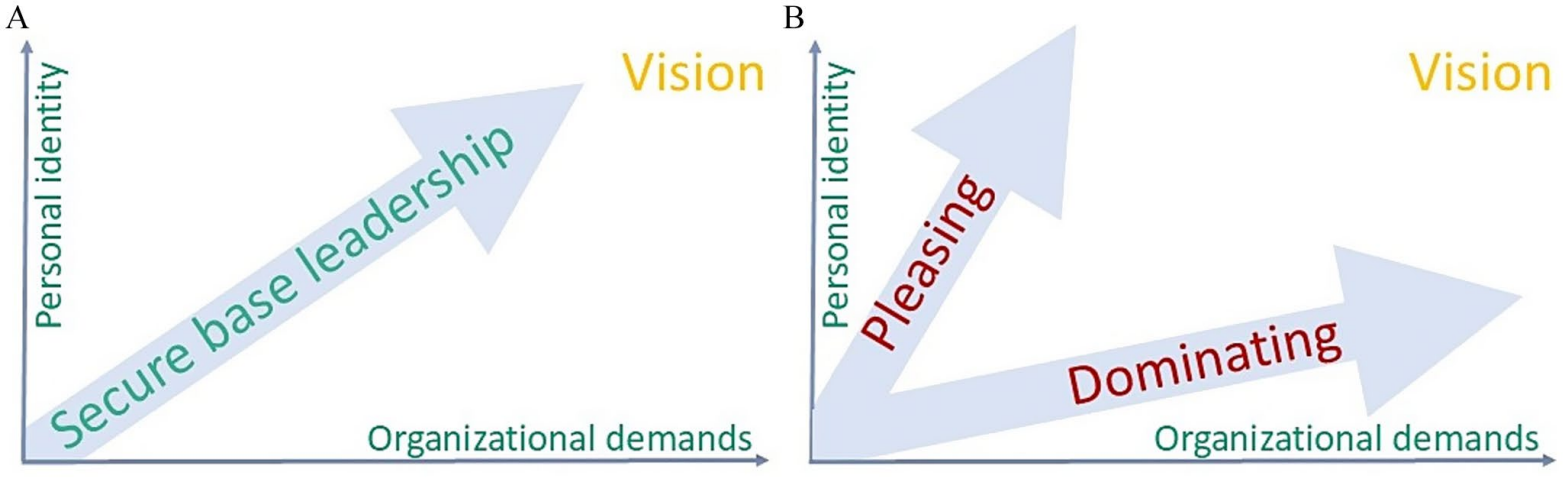
Secure base leadership as an integrated “care to dare”. **(A)** When managed well over time, this balance between care and challenge unlocks untapped potential. **(B)** The dynamic nature over time, from single moments to longer periods, is key: when organizational values or goals are at risk, a secure-base leader reinforces means of daring. Contrary, when individuals or teams struggle for any given reason, reinforcement of caring has to become a leader’s integral duty. Yet, key is to strive for the well-managed balance over time to foster a culture of secure base leadership and avoid the development of a culture of domination or pleasing arising from situational leadership behavior.

Situated in relation to established leadership theories, secure base leadership (Kohlrieser et al., 2012) can be captured with the words “caring” and “daring.” By embedding secure base leadership within this landscape of leadership theories, we can see it as both complementary to, and distinct from, existing models: it uniquely integrates the care-dare balance with psychology and links leadership behaviors to personal growth. Addressing this embodiment of leadership-related “being” to resolve the paradoxical tension between individual and organizational needs, the concept of secure base leadership is a powerful exploration of how leaders can inspire exceptional performance by being both caring and challenging (Kohlrieser et al., 2012). The concept blends the intertwined combination of emotional support (“caring”) with the courage to push individuals and teams beyond their comfort zones (“daring”) to enable expansion of human potential. The term *secure base* originates from attachment theory, first developed by British psychologist John Bowlby in the mid-20th century. Bowlby used the term to describe the role of a caregiver - typically a parent - who provides a reliable foundation of safety and support from which a child can explore the world. The idea was later expanded by developmental psychologist Mary Ainsworth, whose research demonstrated that children with a secure attachment were more confident in exploring their environment, knowing they could return to a trusted figure for comfort and reassurance (Ainsworth, 1979). A secure base provides a sense of safety and trust, enabling individuals to take risks and grow. By creating such a secure environment and additionally daring others to excel, leaders can cultivate high-performing, resilient individuals, teams and organizations (Figure 1A) (Kohlrieser et al., 2012).

Secure base leadership requires a dynamic and proactive management of “caring” and “daring” over time: when organizational values or goals are at risk, a secure-base leader reinforces means of daring. Contrary, when individuals or teams struggle for any given reason, reinforcement of caring must become a leader’s integral duty. If, however, these forces are inappropriately balanced over a longer period, leadership may become, in Bowlby caregiver logic, unfruitful patriarchic domination or mothering pleasing (Figure 1B).

## The “right theory”

Despite the alleged diversity of global cultures and differences in rituals or dogma, key concepts are shared across different cultures uniting them around common themes of subjective experience and morality and reflect humanity’s universal aspirations for purpose and an interconnected existence as part of something greater than oneself (Frankl, 1985). In other words, meaningful answers to presumably most personal questions such as “Who am I? Why am I here? What happens after death?” are universal drivers of personal development and shaped humankind’s understanding of psychology and psychological development of individuals likely for millennia and the debate about the “right theory” was likely entertained for centuries.

### Western perspectives on personal development

Personal development is the key subject of Western 20th century psychological theories. In brief: Freud’s theory focuses on resolving unconscious conflicts at the dualistic Ego stage through practices such as dream analysis (Freud, 1989). Maslow emphasizes the importance of social connection and purpose, aiding growth from the dualistic Ego to transpersonal awareness stages for personal development (Maslow, 2013; Maslow, 1987). Jung’s individuation process supports integration of the shadow and archetypes for authentic self-development (Jung, 2014; Jung and Beebe, 2016).

### Eastern wisdom traditions and leadership

Personal development is also the key subject of Eastern wisdoms. To put it simply, Hindu wisdom advocates self-inquiry (Atma Vichara) and realizing the unity of the individual and universal self (nonduality) (Easwaran, 2007). Buddhist teachings emphasize mindfulness, impermanence, and non-self to dismantle egoic attachments and achieve enlightenment (Hanh, 2013; Kornfield, 2008; Qing, 2017).

Together, these Western and Eastern perspectives provide complementary frameworks for psychological and spiritual growth across different stages of consciousness.

### Aligning leadership with personal development

The developmental trajectory of leadership understanding can be intriguingly mirrored through a biological lens. In 1860, biologist Ernst Haeckel proposed the “recapitulation theory,” famously summarized as *ontogeny recapitulates phylogeny*—the idea that an individual organism’s development reflects the evolutionary stages of its species (Gould et al., 1977). While modern biology has moved beyond the strict literal interpretation of this theory, the metaphor remains powerful: just as embryos pass through stages resembling ancestral forms, leaders often develop through stages that mirror the historical evolution of leadership itself. From primal, survival-based dominance hierarchies to relational, visionary, and purpose-driven leadership, each stage encapsulates the shifting paradigms of human organization. This analogy underscores the value of understanding leadership not as a fixed set of traits, but as an evolving process shaped by both individual growth and cultural context.

### Integral theory as a bridge across traditions

In his influential “Integral theory,” the American philosopher and writer Ken Wilber created a comprehensive framework that tries to integrate science, psychology, spirituality, and philosophy into a unified understanding of human experience and development (Wilber, 1997). Wilber tries to map all dimensions into one big picture. In his book “*No Boundary”* he synthesized an integral framework of the various psychological theories, spiritual traditions, and philosophical perspectives into a unified model of human development and consciousness (Wilber, 2001). According to Wilber, human consciousness exists on a spectrum, ranging from fragmented ego-based awareness to nondual unity consciousness and human suffering and conflict arise from artificial boundaries we create between dualities such as self and others, mind and body, and numerous others (Wilber, 2001). By dissolving these boundaries, individuals can progress toward a unified, holistic sense of being Table 1 provides examples to integrate the contributions of Western psychology (e.g., Freud, 1989; Maslow, 1987; Jung, 2014) and Eastern traditions [Hinduism (Easwaran, 2007), Buddhism (Hanh, 2013; Kornfield, 2008; Qing, 2017; Soeng, 2010)] for personal growth and self-realization.

**Table 1:** Levels of psychological developments and their key characteristics, steps to achieve personal growth, and respective leadership approach.

Level (Wilber, 2001)	Key characteristics	Steps to achieve growth	Leadership approach
Persona-Level	“Doing” (Wilber, 1997; Scharmer, 2016; Senge, 1996; Wilber, 2001) Focus on the “mask” (social self) to identify with socially acceptable roles and to repress unacceptable parts (Jung and Beebe, 2016). Freud’s defense mechanisms operate to protect this level (Freud, 1989). Disconnection from the authentic self, incl. The shadow (Freud, 1989; Jung and Beebe, 2016).	Shadow work: Acknowledge and integrate repressed emotions and aspects of the self through techniques like journaling and therapy (Johnson, 1991). Authenticity practices: Reflect on masks and roles you play to reduce dependence on external validation (Johnson, 1991). Mindfulness: Buddhist Vipassana meditation can help observe and transcend automatic emotional patterns (Hanh, 2013; Wilber, 2001; Kabat-Zinn, 1990).	Create a safe environment where individuals feel valued for their contributions (Empson, 2017; Edmondson, 2018). Help individuals move beyond societal roles and masks to discover their authentic selves (George et al., 2017). Encourage self-reflection and authenticity by providing constructive feedback and recognizing their unique strengths (Zinn, 2017; Kabat-Zinn, 1990; Kaiser et al., 2020). Facilitate the experience of mindfulness, e.g., by recommending mindfulness stress reduction (MBSR) courses (Zinn, 2017; Kabat-Zinn, 1990).
Ego-Level	“Knowing” (Wilber, 1997; Scharmer, 2016; Senge, 1996; Wilber, 2001) Focus on meeting deficiency needs (Maslow, 2013). Awareness of the ego as a central organizing sense of self/“I” (Freud, 1989) Awareness of existential anxieties (Frankl, 1985). Includes the body, which is still experienced as separate (Maslow, 2013). Separation from body and others persists (Maslow, 2013; Freud, 1989).	Logotherapy: Use Frankl’s techniques to identify personal meaning (Frankl, 1985). Body awareness practices: Engage in body scans, yoga or somatic therapies rooted in Hinduism to connect ego with the body (Van der Kolk, 2014). Meditation: Explore mindfulness and concentration techniques rooted in Buddhism to observe the transient nature of egoic thoughts (Kornfield, 2008). Empathy and relational Growth: Recognize how others’ perspectives challenge and expand your sense of self.	Support individuals in integrating their sense of self with their environment, fostering collaboration and resilience (Michel and Neuman, 2010). Support integration of self-awareness with relational dynamics (Michel and Neuman, 2010; Zinn, 2017). Encourage collaborative projects, emphasize the importance of empathy, and foster mindfulness to foster resilience (Zinn, 2017; Kabat-Zinn, 1990; Brown, 2018). Facilitate the experience of body awareness practices, e.g., by supporting MBSR based body scans, yoga courses, etc. (Zinn, 2017; Kabat-Zinn, 1990; Van der Kolk, 2014).
All-Organism	“Being” (Wilber, 1997; Scharmer, 2016; Senge, 1996; Wilber, 2001) Transcendence of the ego and the organism to experience oneness with the universe (Jung and Beebe, 2016; Easwaran, 2007; Soeng, 2010). Buddhist concept of emptiness and non-self (Easwaran, 2007; Hanh, 2013; Kornfield, 2008; Soeng, 2010). Hindu concept of Atman (universal self) as a reflection of Brahman (ultimate reality) (Jung and Beebe, 2016; Easwaran, 2007; Soeng, 2010).	Nondual meditation: Practice Zen meditation or Advaita Vedanta inquiry (e.g., “Who am I?”) to experience the unity of all things (Maharshi, 2015; Salzberg and Kabat-Zinn, 2004). Compassion practices: Extend universal love through Buddhist practices like Metta meditation (Salzberg and Kabat-Zinn, 2004). Mystical texts: Study scriptures like the Bhagavad Gita or Diamond Sutra to deepen the understanding of cosmic unity (Easwaran, 2007; Easwaran, 2011).	Inspire a holistic vision that aligns individual purpose with organizational goals (Luedi, 2022; Laloux, 2014; Wilber, 2001). Inspire teams with a compelling vision that integrates personal and organizational growth (Mandela, 2008). Use coaching mentorship to deepen their connection to a shared mission (Kohlrieser et al., 2012).
All-Unity	“Connecting” (Wilber, 1997; Scharmer, 2016; Senge, 1996; Wilber, 2001) Integration of the self with the body and identification with the environment (Maslow, 1987) (Maslow’s being needs or self-actualization). Recognition of interdependence (Kornfield, 2008; Easwaran, 2007; Wilber, 2001). Increased connection to nature and systems thinking (Easwaran, 2007; Hanh, 2013).	Ecological awareness: Adopt an eco-spiritual perspective to develop a felt sense of connection to nature (Hanh, 2013; Macy, 2021). Systems thinking: Practice seeing relationships as interdependent, aligning with Hindu and Buddhist cosmology (Kornfield, 2008; Wilber, 2001; Peter, 1990). Meditative absorption: Practice spirituality to deepen your experience of interconnection with life (Hanh, 2013; Qing, 2017).	Promote systems thinking and emphasize the interdependence between individuals and their environments (Laloux, 2014). Strategically frame sustainable initiatives and cultivate a sense of purpose that connects personal growth with societal impact (Luedi, 2022; Laloux, 2014). Foster a sense of collaboration and connection to a larger purpose (Luedi, 2022; Laloux, 2014; Scharmer, 2016). Facilitate cross-functional problem-solving by aligning team goals with broader organizational objectives (Luedi, 2022; Laloux, 2014; Scharmer, 2016).

Wilber identifies key stages of psychological and spiritual development, each representing a greater integration of self and a dissolution of perceived separations (Wilber, 2001). Key practices across all stages include self-reflection in the form of journaling, introspection, and questioning one’s beliefs, mindfulness in action to bringing awareness to everyday activities, meditation as a foundation for transcending boundaries at every stage, and compassion to develop empathy for oneself and others.

These practices connect directly with secure base leadership: self-reflection and mindfulness underpin conscious choice, enabling leaders to act intentionally rather than reactively; meditation and the dissolution of ego-derived boundaries provide the inner stability necessary for constructive challenge, allowing leaders to stretch others beyond their comfort zones without inducing fear; and compassion aligns with courageous communication, fostering openness, empathy, and dialogue even in high-stakes contexts. This integration echoes Sri Aurobindo’s Integral Yoga (Aurobindo and Ghose, 1993) and its emphasis on uniting the physical, vital, mental, and spiritual dimensions of human development, which later inspired Wilber’s levels of consciousness (Wilber, 1997). In this way, secure base leadership resonates with both Western developmental psychology and Eastern wisdom traditions, offering a unifying pathway for leaders to embody care and challenge across stages of human growth (Easwaran, 2007; Hanh, 2013; Kornfield, 2008; Qing, 2017; Soeng, 2010; Maharshi, 2015; Easwaran, 2011).

### Secure base leadership across developmental stages

**Figure 2:**
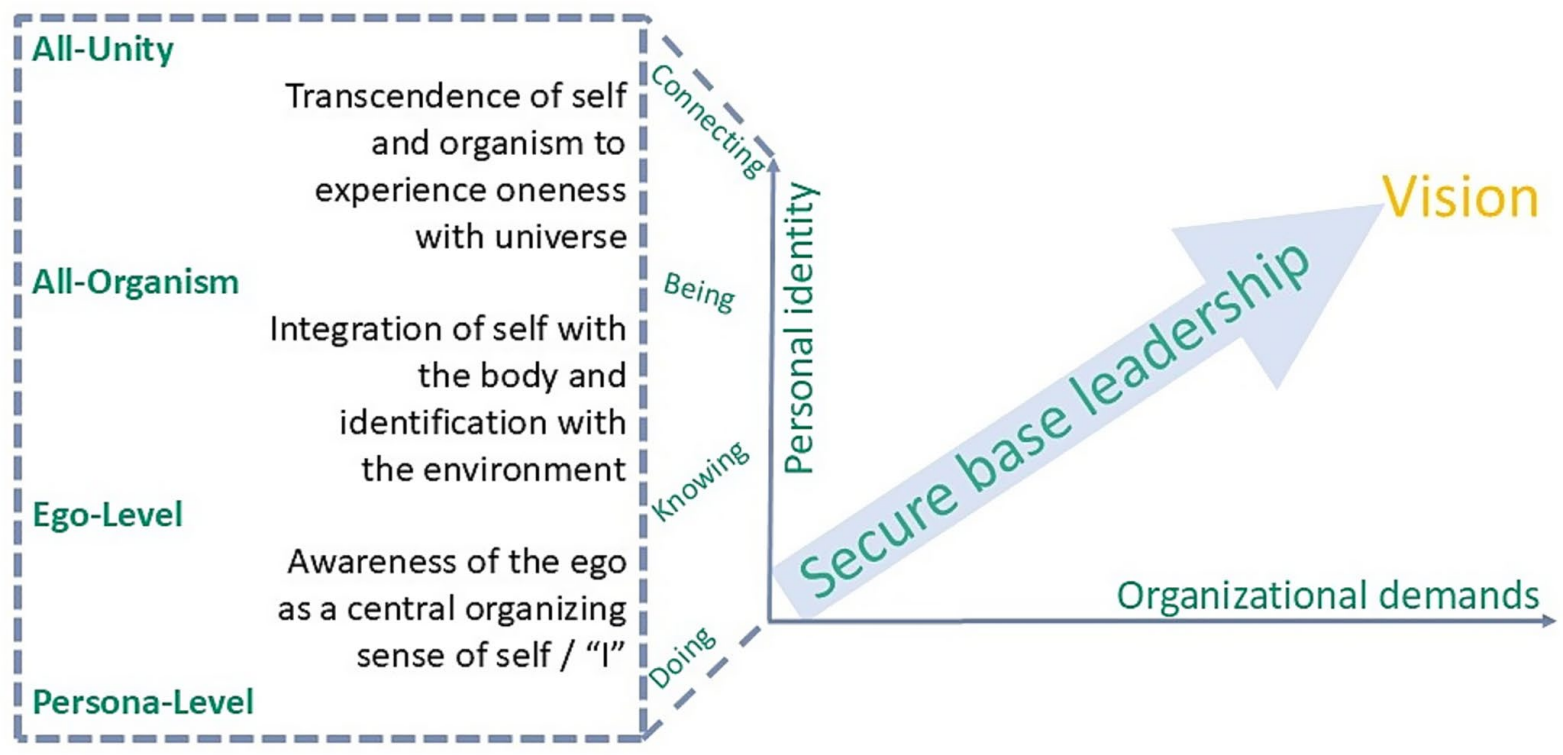
Proposed framework integrating secure base leadership with Wilber’s stages of personal development, ranging from the persona level to the consciousness of all-unity. In many expert organizations, leaders embody “doing” (execution) and “knowing” (intellectual mastery) to sustain productivity and careers, yet an exclusive focus on these dimensions can foster disconnection from the authentic self, burnout, and fragile relationships. Our framework emphasizes balancing “doing” and “knowing” with the less quantifiable but crucial dimensions of “being” (authentic presence and purpose) and “connecting” (trust-based relationships). This verb-based framing aligns with contemporary theories such as Scharmer’s Theory U, Senge’s Learning Organization, and the UN’s Inner Development Goals, underscoring a shift from static traits to dynamic capacities that support sustainable leadership and personal growth.

This integration of Wilber’s developmental stages with leadership approaches underscores the originality of secure base leadership. While authentic, adaptive, transformational, and servant leadership each highlight vital facets of effective leadership, they can be reframed as applied expressions of developmental stages: authentic leadership emerges as leaders move beyond persona-level masks, adaptive leadership reflects ego-level flexibility and learning, transformational leadership resonates with the all-organism stage of shared purpose, and servant leadership embodies the all-unity stage through self-transcendence and service. By explicitly positioning secure base leadership within this developmental spectrum, the actionable framework provides conceptual coherence: it shows how leaders can move between caring and daring behaviors in alignment with their own developmental trajectory and the needs of those they lead. Figure 2 illustrates this synthesis by mapping secure base leadership across Wilber’s stages through the verbs “doing, knowing, being and connecting.” Here our originality lies in linking Wilber’s integrative stages with the care–dare dynamic of secure base leadership. Whereas Wilber maps consciousness, secure base leadership provides the actionable bridge into organizational practice. Developmental psychology grounds this synthesis empirically.

## Making the unconscious conscious: applying secure base leadership in practice

The practical relevance of this synthesis emerges when Secure Base Leadership is explicitly applied through the developmental lens. Rather than treating leadership behaviors as context-free, the model situates them in relation to both the leader’s and followers’ developmental stages. Effective leaders understand that their teams are not static; they are composed of individuals at different stages of personal and professional development. Drawing on a wider perspective of Wilber’s broader integral theory to understand the complexity of human experience not only offers an understanding of individual psychological development in any given cultural context but also provides a link toward secure base leadership of people at different stages of personal development, whereby the individual’s spectrum of consciousness not only develops over time but also depends on current contexts. For effective secure base leadership this implies that you apply a coaching and inspirational leadership style aiming to “make the unconscious conscious” (Jung, 2014) in the person(s) you lead in different contexts at different times. This allows you to address the individual psychological setups in each context and, equally, you are leading people at different stages of personal development (Figure 2). Of note is the fact that both personal and external contexts often impact situational psychological setups and thus behavior and a leader’s responsibility is to seize the right moment to mirror behavior in a coaching style. Applying an inspirational leadership style (embodied being) is similar yet may likely cause an effect in people even when applied in presumably less suitable moments.

## Putting theory into practice: a call to action

**Figure 3:**
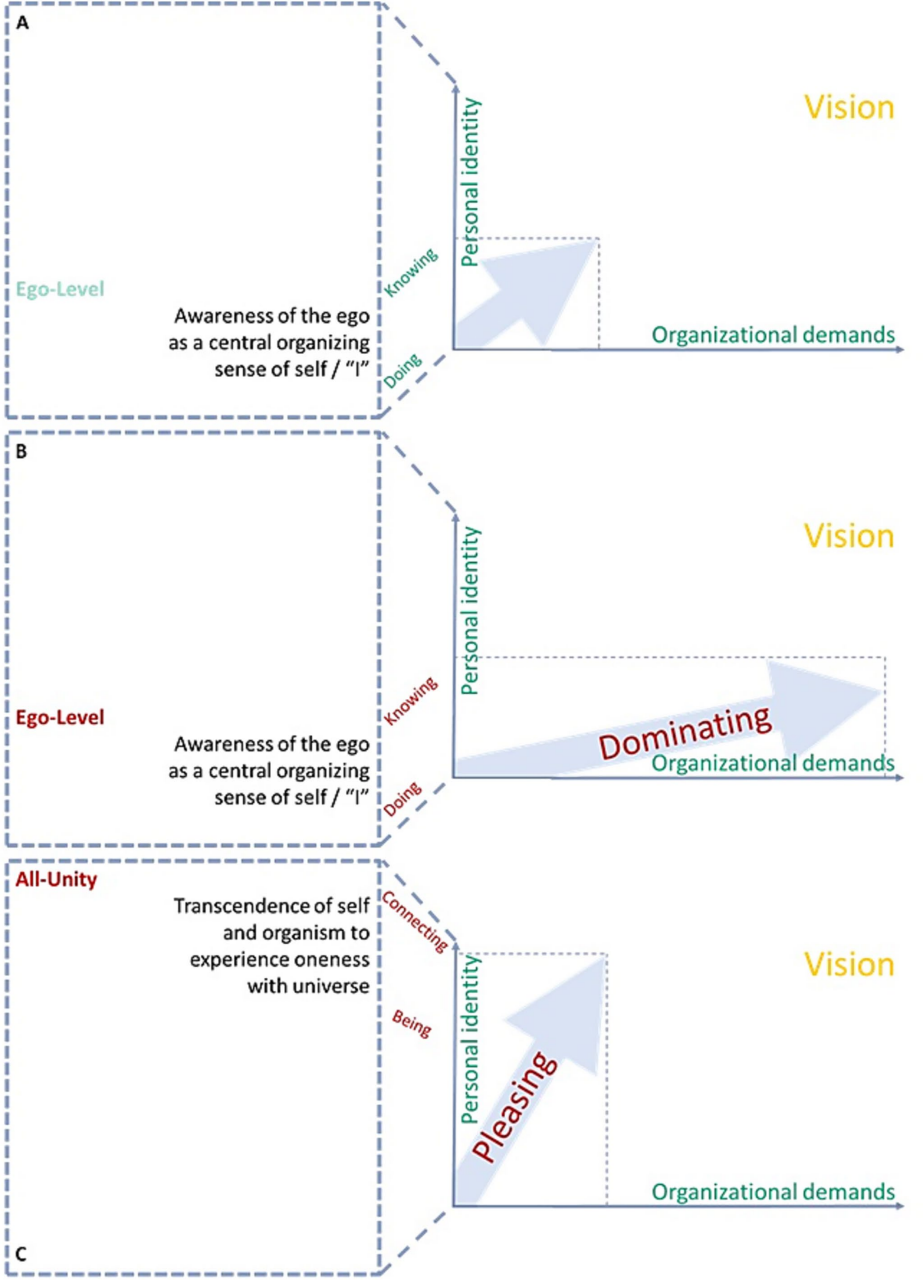
Secure base leadership respecting individual personal development (identity) and organizational demands. **(A)** The identity (e.g., Ego-Level) at equilibrium with the organizational demand from them as professionals. **(B)** The identity (e.g., Ego-Level) in disbalance with the organizational demand from them as professionals whereby the demand exceeds the individual’s personal development, naturally leading to a dominant leadership. **(C)** The identity (e.g., All-Unity) in disbalance with the organizational demand from them as professionals whereby the demand cannot keep up with the individual’s personal development, naturally leading to pleasing leadership.

In the same logic as a leader must reinforce caring when individuals or teams struggle for any given reason or reinforce daring to ensure individuals or teams strive in the best organization’s interest, a secure base needs to ensure their supervised leader’s personal identity is ready for leading the organizational demand before a promotion to a more demanding leadership project. Otherwise, the supervised leaders inevitably display dominant behavior and cannot serve as secure base leaders themselves (or, on the contrary, they risk pleasing their teams, if the organizational setup does not allow them to challenge people). Figure 3 displays respective dimensions with personal identity and organizational demands equilibrated and in disbalance.

### Organizational case example: ALPIQ

To illustrate how secure base leadership works in practice, consider the case of ALPIQ, a leading Swiss energy services provider, which is applying secure base leadership principles to its ongoing cultural transformation. Following an intense leadership experience for the Top 40 in December 2022, the company has embarked on a series of activities, processes and trainings all designed to co-create the conditions where the potential of both people and the organization can be unleashed. More than 30 executives from across all levels of the organization have been trained as “Coaching Ambassadors” in secure base leadership whilst still retaining their ‘daily’ roles. This approach has been taken to embed the theory into daily practice, adapted for the culture, context and readiness stage of specific groups.

This organizational vignette as an illustrative example concretizes our conceptual synthesis by showing how the abstract framework of secure base leadership may translate into leadership action. Like many other organizations working with secure base leadership, the challenge lies in the ‘and’ of both caring and daring. Like many other organizations working with secure base leadership, the challenge lies in the ‘and’ of both caring *and* daring (ALPIQ, 2025). In the words of ALPIQ CEO, Antje Kanngiesser, “*We firmly believe that our company culture is our competitive advantage. To this matter, we align all our actions with our company’s purpose and values. In our complex environment, we consider value-based leadership essential. By both ‘caring’ and ‘daring’, we nurture an environment ripe for perpetual learning and growing and forge secure bases for constructive dialogue and daring decisions”* (ALPIQ, 2025).

### Implications for leadership development programs

While cultures shaped mankind’s understanding of psychology and psychological development of individuals the debate about right leadership (Sachs, 2012; Laloux, 2014), both personal and cultural development do not imply that deeper rooted behavior is obsolete in modern leadership. Likely in any context, there are circumstances when acting from a leadership approach tied to earlier stages of individual or organizational development may be appropriate or more effective than relying mainly, e.g., on embodied being, especially when cultural maturity is lacking: In times of crisis or high-stakes scenarios, swift and authoritative decision-making is often essential. Ego-based instincts, which prioritize clear hierarchy and strong leadership, can bring order and direction during chaos. For example, a leader may need to take decisive action to ensure the safety of a team during a sudden organizational or external emergency (Laloux, 2014). New or unstable teams often require a foundational level of trust and cohesion before transitioning to a higher level of development. In such cases, fostering a sense of belonging, loyalty, and shared purpose hallmarks of tribal instincts, can help establish a stable base. For instance, a leader might initially rely on clear authority (knowing and doing) to align a team before moving toward distributed decision-making (Laloux, 2014).

## Future directions and research opportunities

Future research should empirically examine the integrative hypotheses presented here by combining longitudinal and multi-method approaches. For example, studies could investigate whether leaders trained in secure base leadership demonstrate measurable improvements in team outcomes such as psychological safety, resilience, and performance across different stages of personal development. Experimental or quasi-experimental designs could compare cohorts exposed to secure base interventions with control groups, while mixed-method case studies may capture the lived experience of leaders and followers in diverse organizational contexts. Such empirical work would strengthen the validity of the proposed conceptual synthesis and provide evidence-based pathways for embedding secure base leadership across different stages of personal development into leadership development programs.

## Toward conscious and intentional leadership

In a world marked by accelerating complexity and uncertainty, leadership is no longer a role—it is about a conscious way of being. Secure base leadership offers a transformative path by honoring where people are in their personal development while daring them to grow beyond it. By integrating psychological insight, emotional intelligence, and timeless spiritual wisdom, leaders can transcend transactional management and cultivate resilient, purpose-driven individuals and teams. The essence of this approach lies in presence—in choosing to lead not from fear or ego, but from awareness and intention.

Taken together, situating secure base leadership alongside contemporary leadership theories while grounding it in developmental psychology and integral theory strengthens its conceptual coherence. Whereas authentic, adaptive, transformational, and servant leadership each provide valuable perspectives, secure base leadership synthesizes their insights into a unified framework that emphasizes the dynamic equilibrium of caring and daring. Its contribution lies in bridging theory and practice: it connects the developmental stages of human growth with practical leadership behaviors, offering a coherent pathway for leaders to honor individual identity while meeting organizational demands. In doing so, we do not replace other models but rather situate secure base leadership firmly within contemporary leadership studies while offering an integrative, developmentally grounded framework that advances the field.

In the words of Viktor Frankl, “Between stimulus and response lies a space. In that space lies our power to choose our response. In our response lies our growth and our freedom” (Frankl, 1985). Neuroscience research provides compelling evidence for Frankl’s assertion about the space between stimulus and response. Studies on neural response timing have demonstrated that there exists approximately 0.2 s between when a stimulus reaches our sensory system and when our autonomic response begins (McFarland and Goldsworthy, 2013). This brief window represents the neurological basis for conscious intervention in otherwise automatic reactions. For secure base leaders, this 0.2-s gap is where conscious leadership occurs—the space where they can choose care over reaction or challenge over complacency (McFarland and Goldsworthy, 2013). By developing awareness of this neurological window, leaders can cultivate the capacity to respond intentionally rather than reactively, embodying the balance between care and dare that defines secure base leadership.

The contribution of this paper is not breadth of references, but depth of synthesis. By explicitly integrating developmental psychology, Integral Theory, and secure base leadership, we provide a coherent framework that connects personal growth with leadership practice. This integration sharpens the originality of secure base leadership as a model that honors individual development while meeting organizational demands.

This neurobiological understanding aligns with our evolutionary development. Our brain stem which controls fear and aggression is the oldest and thus strongest part of the brain. In the evolution from vertebrates toward mammals, the limbic system, controlling emotional judgement (and in humans consecutively dualistic thinking), came along (Porges, 2003). And, finally, primates were gifted with an amazing cerebrum that allows abstract thinking. Thereby, humans have the possibility to control their mind and thereby possess the gift of choosing their mental setup that also allows for personal development (Michel and Neuman, 2010; Jung, 2014; Kornfield, 2008; Zinn, 2017). Leaders who succeed in the embodiment of Frankl’s wisdom and act from this space are a secure base for themselves, act consciously, and lead intentionally even in highly demanding environments, thereby supporting others as a secure base at different stages of personal development.

By recognizing the neurobiological basis for conscious intervention and embracing a more participatory view of human consciousness, secure base leaders can create conditions for both individual flourishing and organizational excellence. This integration of ancient wisdom with cutting-edge science offers a path forward for leadership that is both deeply human and profoundly effective in our complex world.

## Data Availability

The original contributions presented in the study are included in the article/supplementary material, further inquiries can be directed to the corresponding author.

## References

[ref1] Ainsworth M. S. (1979). Infant–mother attachment. Am. Psychol. 34, 932–937. doi: 10.1037/0003-066X.34.10.932, PMID: 517843

[ref2] ALPIQ. Our leadership principles. (2025). Available online at: https://wwwalpiqcom/about-us/our-purpose/our-leadership-principles?utm_source=chatgptcom2025 (Accessed April 5, 2025).

[ref3] Ankrah D. Bristow J. Hires D. Artem Henriksson J. (2023). Inner development goals: from inner growth to outer change. Field Actions Sci. Rep. J. Field Actions, 82–87.

[ref4] Aurobindo S. Ghose A. (1993). The integral yoga: Sri Aurobindo's teaching and method of practice: Selected letters of Sri Aurobindo. Lohne (Germany): Lotus Press.

[ref5] Avolio B. J. Gardner W. L. (2005). Authentic leadership development: getting to the root of positive forms of leadership. Leadersh. Q. 16, 315–338. doi: 10.1016/j.leaqua.2005.03.001

[ref6] Bass B. M. Riggio R. E. (2006). Transformational leadership. New York: Psychology press.

[ref7] Brown B. (2018). Dare to lead: Brave work. Tough conversations. Whole hearts. New York: Random house.

[ref9] Easwaran E. (2007). The upanishads. Tomales, CA: Nilgiri Press.

[ref10] Easwaran E. (2011). Essence of the Bhagavad Gita: A contemporary guide to yoga, meditation, and Indian philosophy. Tomales, CA: Nilgiri Press.

[ref11] Edmondson A. C. (2018). The fearless organization: Creating psychological safety in the workplace for learning, innovation, and growth. Hoboken, NJ: John Wiley & Sons.

[ref12] Empson L. (2017). Leading professionals: Power, politics, and prima donnas. Oxford: Oxford University Press.

[ref13] Frankl V. E. (1985). Man's search for meaning. New York: Simon and Schuster.

[ref14] Freud S. (1989). The ego and the id (1923). TACD J. 17, 5–22. doi: 10.1080/1046171X.1989.12034344

[ref15] George B. (2003). Authentic leadership. San Francisco: Jossey-Bass.

[ref16] George B. Ibarra H. Goffee R. Jones G. (2017). Authentic leadership. Boston: Harvard Business Review Press.

[ref17] Goleman D. Leadership that gets results. Leadership perspectives. Abingdon-on-Thames, UK: Routledge; 2017:85–96.

[ref18] Gould S. J. Gould S. J. Gould S. J. Gould S. J. (1977). Ontogeny and phylogeny. Cambridge, MA: Belknap Press of Harvard University Press.

[ref19] Greenleaf R. K. (1998). The power of servant-leadership. Oakland, CA: Berrett-Koehler Publishers.

[ref20] Hanh T. N. (2013). Love letter to the earth. Berkeley, CA: Parallax Press.

[ref21] Heifetz R. A. (1994). Leadership without easy answers. Boston, MA: Harvard University Press.

[ref22] Johnson R. (1991). Owning your own shadow: Understanding the dark side of the psyche. San Francisco: Harper.

[ref23] Jung C. G. (2014). Aion: Researches into the phenomenology of the self. Abingdon-on-Thames, UK: Routledge.

[ref24] Jung C. Beebe J. (2016). Psychological types. Abingdon-on-Thames, UK: Routledge.

[ref25] Kabat-Zinn J. (1990). Full catastrophe living: Using the wisdom of your body and mind to face stress, pain, and illness. Brooklyn, NY: Delta.

[ref26] Kaiser H. A. Peus M. Luedi M. M. Lersch F. Krejci V. Reineke D. . (2020). Frontal electroencephalogram reveals emergence-like brain activity occurring during transition periods in cardiac surgery. Br. J. Anaesth. 125, 291–297. doi: 10.1016/j.bja.2020.05.064, PMID: 32682555

[ref27] Kohlrieser G. Goldsworthy S. Coombe D. (2012). Care to dare: Unleashing astonishing potential through secure base leadership. Hoboken, NJ: John Wiley & Sons.

[ref28] Kornfield J. (2008). The Dhammapada: Teachings of the buddha. Boulder, CO: Shambhala Publications.

[ref29] Laloux F. (2014). Reinventing organizations. Brussels: Nelson Parker.

[ref30] Linsky M. Heifetz R. A. (2002). Leadership on the line. Boston, MA: Harvard Business School.

[ref31] Luedi M. M. (2022). Leadership in 2022: a perspective. Best Pract. Res. Clin. Anaesthesiol. 36, 229–235. doi: 10.1016/j.bpa.2022.04.002, PMID: 36116904

[ref32] MacGregor B. J. (1978). Leadership. New York: Torchbooks.

[ref33] Macy J. (2021). World as Lover, World as Self: Courage for Global Justice and Planetary Renewal. Parallax Press.

[ref34] Maharshi S. R. (2015). Nan Yar–Who am I? I Sri Ramana Maharshi I Spiritual Classic I Advaita Vedanta. Austin, TX: Open Sky Press.

[ref35] Mandela N. (2008). Long walk to freedom: The autobiography of Nelson Mandela. Hachette, UK.

[ref36] Maslow A. H. (1987). Motivation and personality. London: Pearson Education India.

[ref37] Maslow A. H. (2013). Toward a psychology of being. New York: Simon and Schuster.

[ref38] McFarland W. Goldsworthy S. (2013). Choosing change: How leaders and organizations drive results one person at a time. Columbus, Ohio: McGraw Hill Professional.

[ref39] Michel J. E. Neuman A. (2010). Positive psychology as a catalyst for change. Harv. Bus. Rev.

[ref40] Peter S. (1990). The fifth discipline. The art & Practice of learning organization. New York: Doupleday Currence.

[ref41] Porges S. W. (2003). The polyvagal theory: phylogenetic contributions to social behavior. Physiol. Behav. 79, 503–513. doi: 10.1016/S0031-9384(03)00156-2, PMID: 12954445

[ref42] Qing M. (2017). The Buddhist psychological concept of Paticcasamuppāda. 3rd International Conference on Humanity and Social Science (ICHSS 2017).

[ref43] Russell B. (2004). History of western philosophy. Abingdon-on-Thames, UK: Routledge.

[ref44] Sachs J. (2012). Winning the story wars: Why those who tell (and live) the best stories will rule the future. Boston: Harvard Business Press.

[ref45] Salzberg S. Kabat-Zinn J. (2004). Lovingkindness: The revolutionary art of happiness. Boulder, CO: Shambhala Publications.

[ref46] Scharmer C. O. (2016). Theory U: Leading from the future as it emerges. Oakland, CA: Berrett-Koehler Publishers.

[ref47] Senge P. M. (1996). Leading learning organizations. Training Development 50, 36–37.

[ref48] Smith H. (2009). The world's religions (plus). New York, NY: HarperOne.

[ref49] Soeng M. (2010). The heart of the universe: Exploring the heart sutra. New York: Simon and Schuster.

[ref8] Van der Kolk B. (2014). The body keeps the score: Brain, mind, and body in the healing of trauma. New York.

[ref50] Wilber K. (1997). An integral theory of consciousness. J. Conscious. Stud. 4, 71–92.

[ref51] Wilber K. (2001). No boundary: Eastern and Western approaches to personal growth. Boulder, CO: Shambhala Publications.

[ref52] Zinn J. K. (2017). Mindfulness for beginners. Mumbai: Jaico Publishing House.

